# Implementing pictorial health warning labels on waterpipe tobacco products: a qualitative study

**DOI:** 10.1093/eurpub/ckac131.551

**Published:** 2022-10-25

**Authors:** R Nakkash, M Jaafar, T Asfar, S Chehab, W Maziak

**Affiliations:** 1 Health Promotion and Community Health Department, American University of Beirut, Beirut, Lebanon; 2 Global and Community Health Department, George Mason University, Fairfax, USA; 3 Department of Public Health Sciences, University of Miami, Miami, USA; 4 Epidemiology Department, Florida International University, Miami, USA

## Abstract

**Background:**

Waterpipe smoking rates in Lebanon are among the highest in the in the world. Research has documented the effectiveness of introducing pictorial health warning labels (PHWLs) in curbing waterpipe smoking. Seventeen years after ratification of the WHO Framework Convention on Tobacco Control and twelve years post adoption of a tobacco control law, PHWLs have not yet been implemented in Lebanon. This study aims to gain insight into stakeholders opinions and recommendations for adopting and implementing PHWLs on WP products within the current tobacco control policy environment.

**Methods:**

We conducted 13 online interviews with policymakers, media, owners of establishments that serve waterpipe, as well as international and local NGO representatives whose mandate is tobacco control. During the interview process, key informants were shown PHWLs on waterpipe tobacco products and asked about feasibility of implementation and enforcement. National documents and legislations related to PHWLs were obtained from public record. We conducted content analysis on the documents. Interviews were transcribed, coded, and analyzed thematically.

**Results:**

The majority of the key informants agreed on who is responsible for enforcement of PHWLs and on the contextual obstacles to enforcement. Main barriers to implement PHWLs on WP products were: 1) the fact that the WP is a multi-component tobacco use method that will require including all WP components (charcoal, tobacco, device); 2) WP usually is used in several locations (e.g., home, restaurants); and 3) WP is sold via multiple sources (supermarkets, tobacco shop, etc). Stakeholders recommended some solutions to address barriers to implementation.

**Conclusions:**

Stakeholders responsible for implementation of PHWLs need to take into consideration contextual barriers as well as the particularities of waterpipe tobacco smoking in terms of multiple components used to smoke, locations of consumption, and sources where it is sold.

**Key messages:**

• Implementing PHWLs on waterpipe tobacco products requires distinct understanding of policy environment and context.

• Implementing PHWLs on waterpipe tobacco products needs to address the complex nature of waterpipe smoking as a multi-component tobacco use method .

